# First record of a freshwater bryozoan species in Cuba: *Plumatella repens* (Linnaeus, 1758) (Phylactolaemata, Bryozoa)

**DOI:** 10.3897/zookeys.918.38665

**Published:** 2020-03-12

**Authors:** Rafael Carballeira, Cosme D. Romay, Atocha Ramos

**Affiliations:** 1 Departamento de Ciencias da Terra, Facultade de Ciencias, Universidade da Coruña, Campus da Zapateira, 15071 A Coruña, Galicia, Spain Universidade da Coruña A Coruña Spain; 2 Grupo de Investigación en Bioloxía Evolutiva (GIBE), Departamento de Bioloxía, Facultade de Ciencias, Universidade da Coruña, Campus da Zapateira 15071 A Coruña, Galicia, Spain Universidade da Coruña A Coruña Spain; 3 Grupo de investigación Química Analítica Aplicada (QANAP), Departamento de Química, Facultade de Ciencias, Universidade da Coruña, Campus da Zapateira 15071 A Coruña, Galicia, Spain Universidade da Coruña A Coruña Spain; 4 Centro de Investigacións Científicas Avanzadas (CICA), Universidade da Coruña, As Carballeiras, s/n, Campus de Elviña, 15071 A Coruña, Galicia, Spain Universidade da Coruña A Coruña Spain

**Keywords:** Caribbean Islands, Cuba, floatoblast, Phylactolaemata, salinity, water chemistry

## Abstract

The discovery of *Plumatella repens* floatoblasts in wetlands of the La Niña Bonita Reservoir and the Ciénaga de Zapata Swamp, Cuba, constitutes the first record of a freshwater bryozoan species on the island and extends the distribution range of the species in the insular Caribbean. Unlike the inland waters of the Lesser Antilles the greater availability of water and lower salinity are likely the main factors that determine the distribution of *P. repens* in the Greater Antilles.

## Introduction

The freshwater bryozoan fauna of the insular Caribbean has been mainly studied in the Leeward Islands (Aruba, Bonaire, Klein Bonaire and Curaçao), with three known species from the study of colonies and floatoblasts (statoblast buoyant with the annulus composed of gas chambers): *Plumatella agilis* (Marcus, 1942), *Plumatella casmiana* Oka, 1907 and *Plumatella longigemmis* Annandale, 1915 (Lacourt 1955, 1968); the latter was also reported in Jamaica (Lacourt 1968). In addition, *Plumatella repens* (Linnaeus, 1758) has been reported in Puerto Rico (Osburn 1940; Rogick and Brown 1942; Lacourt 1968). Unidentified *Plumatella* colonies and floatoblasts were reported on the islands of Cuba and Trinidad (Osburn 1940; Rogick and Brown 1942; Lacourt 1968; Collado et al. 1984).

Knowledge of the distribution of freshwater bryozoans in the Caribbean is scarce despite the great biogeographical interest of this area. It constitutes a complex island system located between two large continental biogeographic regions: Nearctic and Neotropical (Wood 2002; Massard and Geimer 2008a, 2008b). New records of *P. repens* from floatoblasts on the island of Cuba contribute to the understanding of the ecology and distribution of freshwater bryozoan species in the Caribbean Islands.

## Material and methods

### Study area

**Figure 1.**
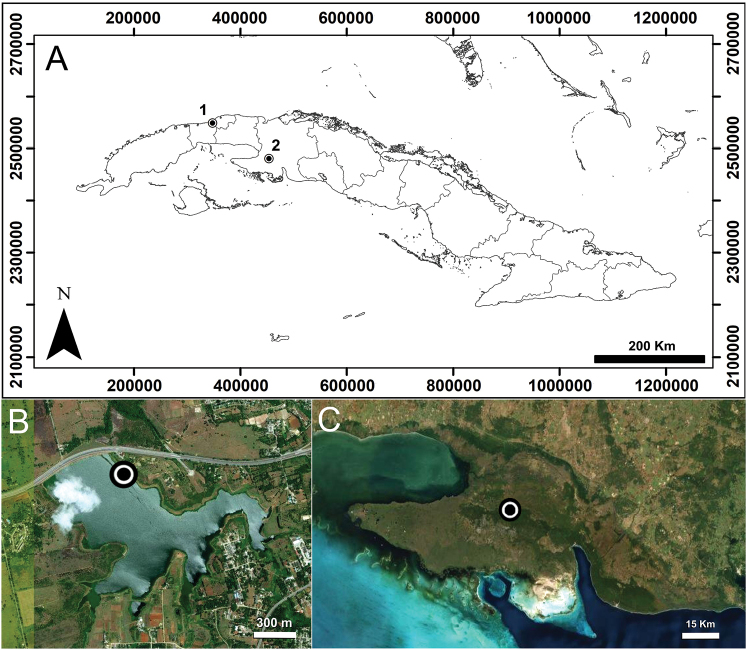
Presence of *Plumatella repens* (Linnaeus, 1758), in the wetlands of Cuba: **A** Location map of localities (1) La Niña Bonita Reservoir and (2) Ciénaga de Zapata Swamp **B** Aerial photograph of the La Niña Bonita Reservoir (ESRI World Imagery, ArcGIS 10.0) **C** Aerial photograph of the Ciénaga de Zapata Swamp (ESRI World Imagery, ArcGIS 10.0).

The La Niña Bonita Reservoir is located in the council of Bauta (Artemisa Province, Cuba) (Fig. 1a, b). This water body is a freshwater wetland with an area of 1.20 ha and a maximum depth of 10 m. This reservoir dams the Jaimanitas River, with a basin of 9.2 km^2^ dominated by limestone rocks, and is used mainly for irrigation and fish farming (Valdés et al. 1996).

Ciénaga de Zapata Swamp is located in the Zapata Peninsula (Matanzas Province, Cuba) (Fig. 1a, c). This wetland is the largest (2600 km^2^) and best conserved marsh swamp in the insular Caribbean, as well as the one with the greatest biodiversity. It has been declared a national conservation area by the government of Cuba and has been internationally recognized as a Ramsar Site (http://www.snap.cu/index.php/ct-menu-item-15/ct-menu-item-67/ct-menu-item-68). The shallow marshes show an important accumulation of organic matter and the lithology is dominated by limestones and dolomites with seeping underground waters (cenotes) (Ferrera et al. 1996; Morell et al. 1997). The waters are bicarbonated-calcic with a great spatial heterogeneity depending on the input of groundwater seeps or marine intrusions. Also, there is a great salinization of groundwater as a consequence of the exploitation of freshwater aquifers (Fagundo et al. 1992b; Rodríguez et al. 1992; Ferrera et al. 1996, 1999; Molerio-León and Parise 2008).

### Sample collection and processing

In shallow wetland areas, samples of 2 cm^3^ of surface sediment were collected in the La Niña Bonita Reservoir (23°02'24.53"N, 82°29'37.09"W; 42 m a.s.l.) (Fig. 1b) and in the Ciénaga de Zapata Swamp (22°25'44.55"N, 81°27'25.26"W; 5 m a.s.l.) (Fig. 1c). Sediment samples were screened through a 50 μm mesh; the larger fractions were examined under a stereoscopic microscope, and floatoblasts were collected with a pipette. Floatoblasts were treated with 2% NaOH for 1 min under agitation at room temperature, then subjected to an ultrasonic bath for 15 seconds, and finally washed in deionized water. Floatoblasts for scanning electron microscopy (SEM) were mounted on aluminium stubs, sputtered with platinum/palladium (15 nm) for 1 min using a Cressington Sputter Coater 208HR SEM, and studied with a JEOL Field Emission SEM JSM 7200F operated at 15 kV in the University of A Coruña’s Research Support Service (Servizo de Apoio a Investigación, S.A.I.).

## Results

**Figure 2.**
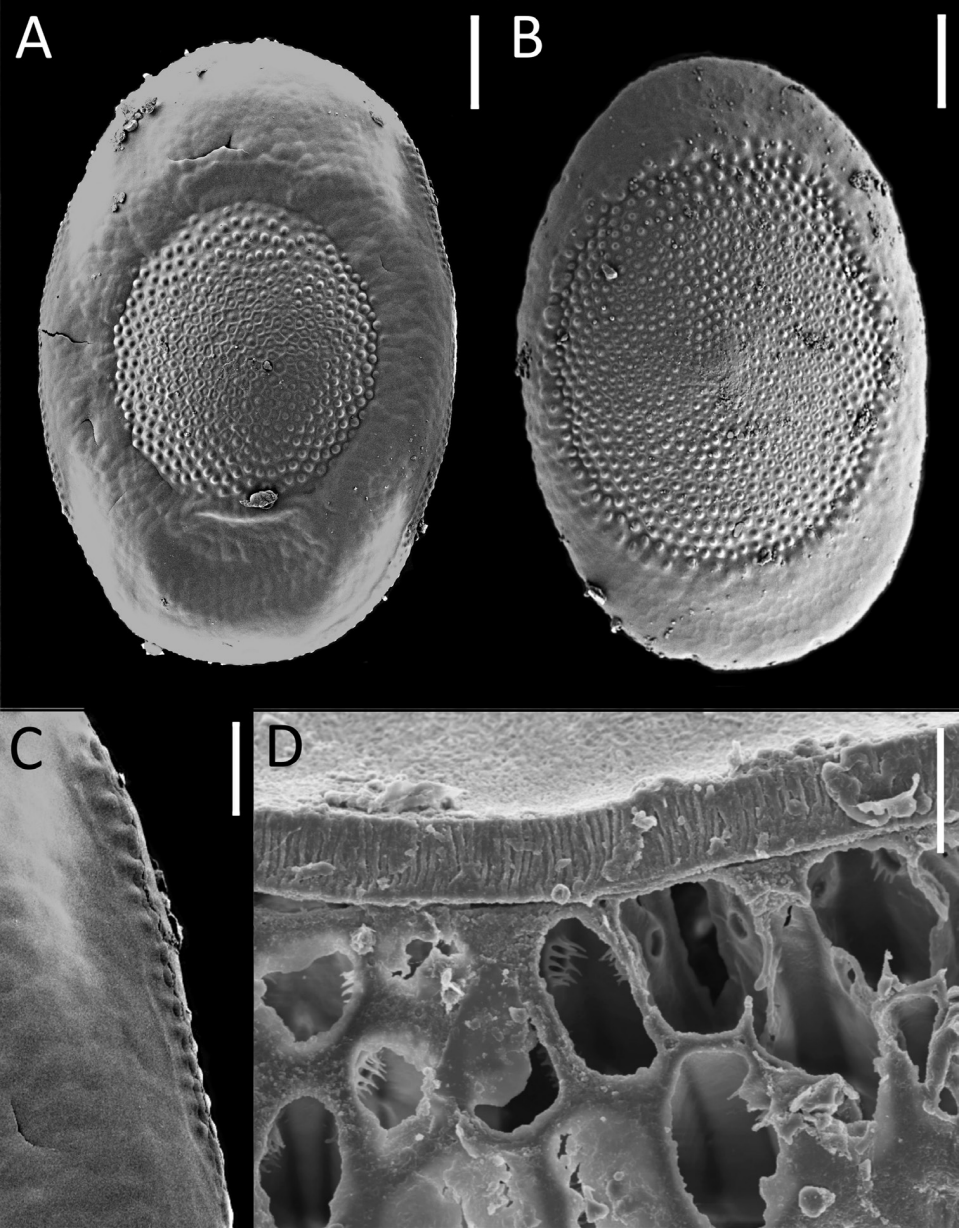
*Plumatella repens* (Linnaeus, 1758), floatoblast from La Niña Bonita Reservoir and the Ciénaga de Zapata Swamp (Cuba), SEM: **A** View of dorsal valve **B** View of ventral valve **C** Suture between valves is a single cord with a row of low tubercles on either side **D** Section of the annulus showing the connection between gas chambers, with circular pores with filiform extensions along the border. Scale bars: 50 µm (**A**, **B**); 10 µm (**C**); 5 µm (**D**).

The morphometry of the examined floatoblasts showed that they belong to the species *Plumatella repens*. The shape of the floatoblast is broadly oval, both valves are equally convex in lateral view, and the floatoblast annulus is smooth, without tubercles (Fig. 2a, b). Floatoblast measurements were 332.2 ± 14.11 (318.5–350.2) μm in total length and 220.8 ± 15.42 (217.3–252.6) μm in total width (*N* = 10). The fenestra of floatoblasts is rounded oval in dorsal view and oval in ventral view, covered with rounded tubercles and a relatively intense reticulation (Fig. 2a, b).

The length of the dorsal fenestra is larger than half the total length of the floatoblast. The annulus is smooth, without tubercles, occasionally with moderate nodulation and some large tubercles on the periphery, around the fenestrae especially on the ventral side (Fig. 2a, b). The measurements of the dorsal fenestra are 158.3 ± 12.40 (130.9–176.4) μm in length and 137.5 ± 10.18 (105.5–163.7) μm in width, while the ventral fenestra measures 216.4 ± 14.56 (187.5–247.3) μm in length and 179.2 ± 12.45 (141.2–188.7) μm in width. The suture between the valves is a single cord with tubercles on both sides (Fig. 2c). A section of the annulus shows circular pores with filiform projections connecting the gas chambers (Fig. 2d).

## Discussion

**Figure 3.**
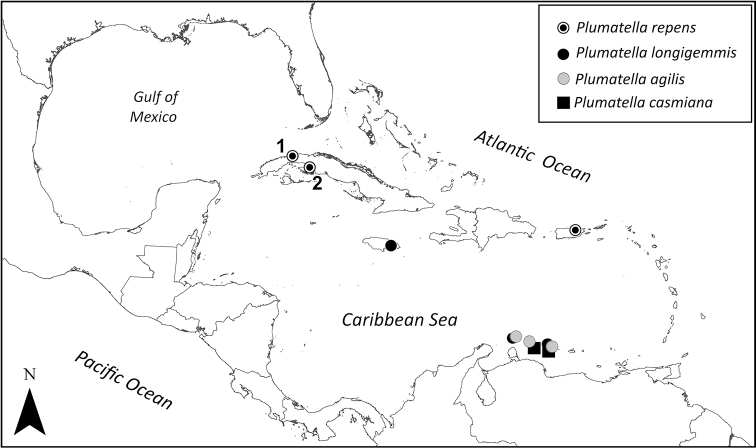
Geographical distribution of *Plumatella* species in the insular Caribbean area, indicating the new records of *P. repens* in Cuba (**1**) La Niña Bonita Reservoir and (**2**) Ciénaga de Zapata Swamp.

These new records of *P. repens* are the first certain record of a freshwater bryozoan species in Cuba; only *Plumatella* sp. was reported on the island previously, without specifying a locality (Collado et al. 1984). This extends the distribution range of the species in the Caribbean area (Table 1; Fig. 3), with a single record in Puerto Rico so far (Osburn 1940; Rogick and Brown 1942; Lacourt 1968). The presence of *P. repens* on the island of Cuba is consistent with the existence of records of the species in the insular Caribbean and the cosmopolitan distribution of this species (Wood 2002; Massard and Geimer 2008a, 2008b).

**Table 1.** Records of *Plumatella* species in the Caribbean Islands area including a description of the localities.

**Species**	**Locality**	**Island**	**Reference**
*Plumatella agilis* (Marcus, 1942) (= *Hyalinella agilis* (Marcus, 1942))	Tanki di Cas Klein St. Joris, rather few algae. Date: 06/09/1936. Chlorinity: 1980 mg Cl l^-1^.	Curaçao	Lacourt (1955, 1968)
	Tanki Monpos, Hato, algae temporary or semi-permanent pools. Date: 11/09/1936. Chlorinity: 310 mg Cl l^-1^.	Curaçao	Lacourt (1955, 1968)
	Pos di Wanga, Middle Curaçao, few algae temporary or semi-permanent pools. Date: 09/11/1936. Chlorinity: 260 mg Cl l^-1^.	Curaçao	Lacourt (1955, 1968)
	Tanki Martha Koosje, Middle Curaçao, some algae temporary or semi-permanent pools. Date: 24/07/1948. Chlorinity: 320 mg Cl l^-1^.	Curaçao	Lacourt (1955, 1968)
	Pos Ariba, Dokterstuin, many algae temporary or semi-permanent pools. Date: 27/10/1937. Chlorinity: 710 mg Cl l^-1^.	Curaçao	Lacourt (1955, 1968)
	Tanki Martha Koosje, Middle Curaçao, some algae temporary or semi-permanent pools. Date: 24/08/1948. Chlorinity: 320 mg Cl l^-1^.	Curaçao	Lacourt (1955, 1968)
	Tanki Leendert, few algae pond, semi-permanent. Date: 16/12/1936. Chlorinity: 35 mg Cl l^-1^.	Aruba	Lacourt (1955, 1968)
	Pos Bronswinkel, overflowing pool, possibly permanent, crowded with algae. Date: 27/03/1937. Chlorinity: 350 mg Cl l^-1^.	Bonaire	Lacourt (1955, 1968)
	Pos Frances, Punt Vierkant, small well in rock crevice, semi-permanent, some algae. Date: 31/03/1937. Chlorinity: 540 mg Cl l^-1^.	Bonaire	Lacourt (1955, 1968)
	Tanki Onima (Sta. 46), on shore of muddy pond, temporary, few algae. Date: 13/11/1936. Chlorinity: 40 mg Cl l^-1^.	Bonaire	Lacourt (1955, 1968)
*Plumatella casmiana* Oka, 1907 (= *Plumatella annulata* (Howata & Toriumi, 1940))	Pos Europa, Dokterstuin, pool, semi-permanent, many algae. Date: 27/10/1936. Chlorinity: 470 mg Cl l^-1^.	Curaçao	Lacourt (1955, 1968)
	Pos di Cas, well, permanent, many algae. Date: 15/11/1936. Chlorinity: 400 mg Cl l^-1^.	Klein Bonaire	Lacourt (1955, 1968)
*Plumatella longigemmis* Annandale, 1915 (= *Hyalinella osburni* (Rogick & Brown, 1942))	Tanki Mon Plaisir, Oranjestad, pool, temporary. Date: 15/12/1936. Chlorinity: 60 mg Cl l^-1^.	Aruba	Lacourt (1955, 1968)
	Tanki di Westpunt, pool, temporary, algae. Date: 09/12/1936. Chlorinity: 80 mg Cl l^-1^.	Aruba	Lacourt (1955, 1968)
	Tanki di Goudmijn Tibushi, Westpunt, puddle, temporary, very few algae. Date: 09/12/1936. Chlorinity: 170 mg Cl l^-1^.	Aruba	Lacourt (1955, 1968)
	Tanki Onima, muddy pond, temporary, few algae. Date: 13/11/1936. Chlorinity: 40 mg Cl l^-1^.	Bonaire	Lacourt (1955, 1968)
	Waterworks of Kingston. Date: 15/06/1946.	Jamaica	Lacourt (1968)
*Plumatella repens* (Linnaeus, 1758)	Stones in the stream Las Piedras.	Puerto Rico	Osburn (1940); Rogick and Brown (1942); Lacourt (1968)
	La Niña Bonita reservoir, freshwater, permanent. Date: 1-12/05/2019. Chlorinity: 66 mg Cl l-^1^.	Cuba	This study
	Ciénaga de Zapata swamp, freshwater to brackish, permanent. Date: 1-12/05/2019. Chlorinity: 305 mg Cl l^-1^.	Cuba	This study
*Plumatella* sp.	Without specify locality	Cuba	Collado et al. (1984)
	Without specify locality	Trinidad	Collado et al. (1984)

The ecology of *P. repens* in Cuba and Puerto Rico is associated with permanent freshwater ecosystems and coastal wetlands with highly mineralized waters caused by the predominant limestone lithology, and influenced by seawater mixing within an oligohaline range (0.5–5‰). The species also experiences a wide range of nutrient concentrations (nitrates, phosphates) and high levels of dissolved organic matter (Ferrera et al. 1996, 1999; Kwak et al. 2007; Molerio-León and Parise 2008).

*Plumatella repens* is present in the La Niña Bonita Reservoir, which has waters of 798 μS cm^-1^ conductivity, pH 8.5, oxic conditions – with dissolved oxygen levels of 80.9 mg l^-1^ – and low concentration of nutrients including both orthophosphates (< 0.10 mg l^-1^) and inorganic nitrogen (< 0.10 mg l^-1^) (Valdés et al. 1996). However, a decrease in water quality in the reservoir was documented during the 1990s due to an increase in organic and sewage discharges (Valdés et al. 1996). The Piedras River in Puerto Rico, where *P. repens* was also recorded (Osburn 1940; Rogick and Brown 1942; Lacourt 1968), is a limestone-dominated basin with 452 μS cm^-1^ conductivity, pH 7.50, and 168 mg l^-1^ total hardness, although with low oxygen concentration (7.49 mg l^-1^), average nitrate levels (0.2 mg l^-1^), and high phosphate levels (0.49 mg l^-1^) derived from organic contamination of anthropogenic origin (Kwak et al. 2007). *Plumatella repens* is also present in the Ciénaga de Zapata Swamp – this coastal wetland has great spatial heterogeneity due to marine intrusion and freshwater springs, and as a result the conductivity range fluctuates between 600 and 2400 μS cm^-1^ from the innermost zones towards the coastal zones (Fagundo et al. 1992a; Rodríguez et al. 1992; Ferrera et al. 1996; Morell et al. 1997).

The conductivity ranges of *Plumatella repens* in the island of Cuba are similar to those documented for continental populations in the north coast of the Gulf of Mexico (Dendy 1963; McCullough and Reed 1987) (Fig. 3), such as Lake Griffin (Florida, U.S.A.), with 290 μS cm^-1^ (Putnam et al. 1972; Taticchi et al. 2009, 2011) and Lake Texoma (Texas, U.S.A.), with 750–1200 μS cm^-1^ (Sublette 1953, 1955, 1957; Gido and Matthews 2000). In addition, populations of P. re*pens* have been recorded in the mouths of the Sabine and Neches rivers, in the coast of Louisiana, in salinities fluctuating between 38 and 4000 μS cm^-1^, sometimes forming small-sized colonies with the brackish-water bryozoan *Victorella pavida* Saville-Kent, 1870 (Wurtz and Roback 1955; Everit 1975; Curry et al. 1981). Similar conductivity ranges have also been documented for *P. repens* in Europe, in the Mediterranean coasts of the Iberian Peninsula, where its presence has been cited in brackish coastal rivers and wetlands with conductivity values reaching up to 2519 μS cm^-1^ and even 3620 μS cm^-1^ (Margalef Mir 1953; Rueda et al. 2001; Rueda et al. 2013; Rueda et al. 2016). Also, Wood and Okamura (2005) mention that *Plumatella repens* can tolerate wide ranges of salinity, from freshwater to oligohaline in the British Isles and continental Europe.

The semi-arid Lesser Antilles, unlike the Greater Antilles, have ephemeral wetlands of small extension, subjected to strong marine salinization and organic discharges due to high anthropic pressure (Van Sambeek et al. 2000; Scalley 2012). Lacourt (1955)'s study on freshwater bryozoan species in the Leeward Islands, despite the scarce ecological data, showed that the temporal stability of aquatic ecosystems and the degree of salinization are factors of great importance for the distribution of freshwater bryozoan species (Fig. 3). In the Leeward Islands, *P. longigemmis* and *P. agilis* inhabit ephemeral pools, while *P. casmiana* is found only in permanent pools (Table 1). *Plumatella longigemmis* appears in freshwater environments with little saline influence (87.5 ± 57.37 (40–170) mg Cl l^-1^), generally under 115 mg Cl l^-1^ (fresh water), while *P. casmiana* is present in waters with mild saline influence (435.0 ± 49.50 (400–470) mg Cl l^-1^), and *P. agilis* is present in waters with a wide range of saline influence (486.5 ± 562.05 (35–1980) mg Cl l^-1^) (Table 1).

*Plumatella repens* is a generalist species with a wide ecological range that can tolerate mild salinization levels; however, the ephemeral nature of these wetlands could constitute the main limitation for its distribution in the Lesser Antilles and could explain the greater affinity between the Greater Antilles and the Nearctic zone of the Gulf of Mexico in the distribution of this species (Fig. 3) (Wood 2002; Massard and Geimer 2008b).

## Conclusions

These new findings of populations of *P. repens* in Cuba constitute the first record of a freshwater bryozoan species on the island, expanding the geographical distribution of this species to the Greater Antilles. The existence of permanent freshwater wetlands in Cuba, unlike in the Lesser Antilles, provides a stable habitat for the species.
